# The Association between COX-2 Polymorphisms and Hematologic Toxicity in Patients with Advanced Non-Small-Cell Lung Cancer Treated with Platinum-Based Chemotherapy

**DOI:** 10.1371/journal.pone.0061585

**Published:** 2013-04-19

**Authors:** Fei Zhou, Guanghui Gao, Shengxiang Ren, Xuefei Li, Yayi He, Caicun Zhou

**Affiliations:** Department of Oncology, Shanghai Pulmonary Hospital, Tongji University School of Medicine, Tongji University Cancer Institute, Shanghai, China; Univesity of Texas Southwestern Medical Center at Dallas, United States of America

## Abstract

**Background and Objective:**

Overexpression of COX-2 is proved to contribute to tumor promotion and carcinogenesis through stimulating cell proliferation, inhibiting apoptosis and enhancing the invasiveness of cancer cells. Apoptosis-related molecules are potential predictive markers for survival and toxicity in platinum treatment. This study aimed at investigating the association between COX-2 polymorphisms and the occurrence of grade 3 or 4 toxicity in advanced non–small cell lung cancer patients treated with platinum-based chemotherapy.

**Materials and Methods:**

Two hundred and twelve patients with inoperable stage IIIB-IV NSCLC received first-line chemotherapy between 2007 and 2009 were recruited in this study. Four functional COX-2 polymorphisms were genotyped by PCR-based restriction fragment length polymorphism (RFLP) methods.

**Results:**

The incidence of grade 3 or 4 hematologic toxicity was significantly higher in G allele carriers of the COX-2 rs689466 (−1195G/A) polymorphism compared with wild-type homozygotes AA (P value = 0.008; odds ratio, 2.47; 95% confidence internal, 1.26–4.84) and the significance still existed after the Bonferroni correction. Statistically significant difference was also found in grade 3 or 4 leukopenia (P value = 0.010; OR = 2.82; 95%CI = 1.28–6.20). No other significant association was observed between genotype and toxicity in the study. The haplotype analysis showed that the haplotype AGG was associated with a reduced risk of grade 3 or 4 hematologic and leukopenia toxicity (P value = 0.009; OR = 0.59; 95%CI = 0.39–0.88 and P value = 0.025; OR = 0.61; 95%CI = 0.39–0.94, respectively) while the haplotype GGG was associated with an increased risk of grade 3 or 4 hematologic and leukopenia toxicity (P value = 0.009; OR = 1.71; 95%CI = 1.14–2.56 and P value = 0.025; OR = 1.65; 95%CI  = 1.06–2.57, respectively).

**Conclusion:**

This investigation for the first time suggested that polymorphism in COX-2 rs689466 may be a potent bio-marker in predicting severe hematologic toxicity in NSCLC patients after platinum-based chemotherapy.

## Introduction

Lung cancer is the most commonly diagnosed cancer and the leading cause of cancer-related death in the world and NSCLC comprises the most common form of it [1]–[2]. Most NSCLC patients diagnosed are in the advanced stages, with the majority of whom presenting with stage III or IV disease. 5-year survival of these patients is still disappointingly low at less than 20% [2].

Platinum-based regimens have been used as the standard first-line chemotherapy in NSCLC patients [3]–[4] while the unpredictable and occasionally serious side effects, especially hematologic toxicity, continue to be an intractable problem. The incidence and severity of toxicities vary greatly between individuals [5]. Thus, searching of predictive markers that can identify patients who will benefit significantly from chemotherapy with minimal toxicity is a necessary and promising job in lung cancer research.

Most platinum compounds induce damage to tumors through induction of apoptosis while apoptosis is responsible for the characteristic hematologic toxicity, gastrointestinal toxicity, and most other drug toxicities [6]. It also suggests that the development of platinum compounds resistance could be the result of either inhibition of apoptotic genes or activation of antiapoptotic genes. Tumors that are resistant to cisplatin might also become resistant to the induction of programmed cell death as a consequence of the development of survival mechanisms during malignant transformation [7]. Therefore, apoptosis-related molecules are potential predictive markers for survival and toxicity in platinum-based treatment. Recently, caspase-3(CASP3), an apoptosis-related gene, was reported to be associated with severe hematologic toxicity risk [6].

Cyclooxygenase-2(COX-2), also known as prostaglandin-endoperoxide synthase 2 (PTGS2), is a key enzyme involved in cancer development and progression and plays an important role in the modulation of apoptosis, angiogenesis, immune response, and tumor invasion [8]–[9]. COX-2 overexpression shows reduced apoptotic susceptibility by up-regulation of Bcl-2 and suppression of CASP3 and CASP9, two important families of apoptosis-related molecules [10]–[11].It is reported that COX-2 is overexpressed in various malignancies such as gastric carcinoma, esophagus carcinoma, including NSCLC, suggesting its involvement in pulmonary tumorigenesis [12]–[14]. Increased COX-2 expression is also associated with more aggressive tumor behavior and poorer prognosis in NSCLC patients [15]. Preclinical study shows that taxanes may stimulate the expression of COX-2 gene and decrease the efficacy of anti-cancer and explain, at least partly, the toxicity of these drugs [16]. Additionally, overexpression of COX-2 mRNA is related to ionizing radiation (IR) induced pulmonary inflammation and inhibiting the IR-induced COX-2 expression could be helpful against radiation-induced normal tissue injury [17].

Several functional single nucleotide polymorphisms (SNPs) that have been identified in the COX-2 gene may contribute to different gene expression or enzyme activities [18]–[19]. A recent study shows that COX-2 gene polymorphism may be a potential predictive marker for survival in locally advanced NSCLC patients treated with chemoradiotherapy or radiotherapy alone [20]. Although the associations between genetic polymorphisms of COX-2 and the risk of developing certain cancers [14], [19], [21], [22] and survival outcome have been reported [20], similar studies with toxicity have rarely been reported.

In this study, four putative functional SNPs in COX-2 gene were investigated. These SNPs include rs689465 (−1290A/G), rs689466 (−1195G/A), and rs20417 (−765G/C) in the promoter region, which were demonstrated to influence the expression of COX-2 [14], [23]; rs3218625 (−1759G/A) in exon10, which was also associated with increased risk of gastric cardia adenocarcinoma [24]. Using DNA samples obtained from a series of patients with advanced NSCLC treated with platinum-based chemotherapy, we assessed the association between these COX-2 polymorphisms and toxicity outcomes.

## Materials and Methods

### Patient recruitment and follow up

Patients with newly diagnosed advanced stage lung cancer were enrolled in the study at Shanghai Pulmonary Hospital in Shanghai, China, between January,2007 and December, 2009. Patients with histological confirmation of NSCLC stage IIIB and stage IV were selected. Other eligibility criteria included Eastern Cooperative Oncology Group performance status (ECOG PS) of 0–2; aged above 18 years; adequate hematologic function (hemoglobin >9 g/dl, neutrophil count >1500/mm^3^, and platelet count>100 000/mm^3^); adequate renal function (creatinine clearance rate >50 ml/sec); adequate liver function (bilirubin <1.5 times the normal upper limit, aspartate aminotransferase and alanine aminotransferase <2 times the normal upper limit) and measurable disease. Patients with symptomatic brain metastases, spinal cord compression, uncontrolled massive pleural effusion and those who previously received chemotherapy were excluded. The protocol was conducted according to the Declaration of Helsinki and Good Clinical Practice guidelines and was approved by the ethics committees of Tongji University Affiliated Shanghai Pulmonary Hospital. The informed consent was written and obtained from each patient before the initiation of any study related procedure. This was a prospectively collection of clinical data and biological samples and later the study was designed.

Clinical data were systematically recorded at entry. Before starting any treatment, all patients underwent a complete medical history interview, physical examination and laboratory testing, including routine hematology and biochemistry analyses, staging with chest radiographs and computed tomography of the thorax and abdomen, and magnetic resonance imaging of the brain and a bone scan.

The incidence of grade 3 or 4 toxicity was assessed twice a week during cycle 1 chemotherapy, then the assessment was repeated once at the beginning of every cycle or before chemotherapy for day 8 treatment according to the National Cancer Institute Common Toxicity Criteria version 3.0 (CTCAE 3.0). Patient charts were reviewed to extract data on toxicities experienced during first-line chemotherapy. The worst toxicity grade of each patient in all the chemotherapy cycles was recorded. The investigators were blinded to the polymorphism status of the patients.

### Chemotherapy Regimens

All 212 patients enrolled in the study were inoperable and were given first-line platinum-based chemotherapy: Cisplatin at a dose of 75 mg/m^2^ or carboplatin (AUC = 5) given on day 1 combined with either gemcitabine at a dose of 1000 mg/m^2^ on days 1 and 8, or vinorelbine 25 mg/m^2^ on days 1 and 8, or paclitaxel 175 mg/m^2^ on day 1, or docetaxel 75 mg/m^2^ on day 1, for a maximum of six cycles, up to disease progression or unacceptable toxicity. The cycles were repeated every three weeks and all chemotherapeutic drugs were administered i.v.

Dose modification was according to the NCCN guideline and it was done by protocol. Briefly, if toxicity higher than grade 3 non-hematology toxicity (except for nausea and vomiting) and grade 4 hematology toxicity, neutropenia without febrile lasting more than 7 days, or febrile neutropenia or infection and/or thrombocytopenia associated with bleeding occurs, the dose of the cytotoxic agents in the next cycle was reduced by 25%. Concomitant supportive therapies, such as erythropoietic agents or granulocyte colony-stimulating factors, were allowed according to the American Society of Clinical Oncology guidelines [25].

### DNA collection and genotyping

Genomic DNA was extracted from 5 mL blood sample that was collected from each patient upon recruitment. Genotypes were determined by PCR-based restriction fragment length polymorphism (RFLP-PCR). The PCR primer pairs used to amplify COX-2 promoter region containing rs689465 (−1290A/G), rs689466 (−1195G/A), rs20417 (−765G/C) and rs3218625 (−1759G/A). The sequences of primers are as followed: 1290F5′-caggttttatgctgtcattttcc-3′/1290R5′ = -tagtgctcagggaggagcat-3′, 1195F5′-ccctgagcactacccatgat-3/1195R5′-gcccttcataggagatactgg-3′, 765F5′-tattatgaggagaatttacctttcgc-3′/765R5′-gctaagttgctttcaacagaagaaat-3′and1759F5′-tgttctcctgcctactggaagc-3′/1759R5′-ctgttgcggagaaaggagtcat-3′.

The primers and reaction conditions were described in our previous report [26]. Genotypes were confirmed by direct DNA sequencing of the PCR products. A 10% blind, random sample of study subjects was genotyped twice by different persons and the reproducibility was 100%.

### Statistics analysis

Toxicity outcome in each group was dichotomized by the presence or absence of grade 3 or 4 toxicity during the first-line treatment. The associations between each genetic polymorphism and grade 3 or 4 toxicity were estimated by odds ratios (OR) and their 95% confidence intervals (95%CI), which were calculated by unconditional logistic regression. Adjusting covariates were performance status, gender, smoking status, histology, stage, radiation therapy, the number of cycles of chemotherapy received during first-line treatment, the receipt of taxanes and the type of platinum of agent (cisplatin versus carboplatin). Tests for trend were done by including genotypes as an ordinal variable in regression models. Hardy-Weinberg equilibrium was tested by Pearson Chi-Square test (*X* ^2^). Allele and genotype frequencies, Hardy-Weinberg equilibrium, linkage disequilibrium analysis, haplotypes and their frequencies were conducted online using SHEsis software platform, which is available at http://analysis.bio-x.cn [27], [28]. For each gene, the Bonferroni correction was made for P value. All P values reported were two-sided, and a level of 0.05 was considered statistically significant. All statistical analyses used SPSS, version 17.0.

## Results

### Patient characteristics and toxicity outcomes

A total of 212 patients with advanced stage NSCLC were enrolled in this study. The median age at diagnosis was 60 years (range, 33–77 years). Of the subjects, 153 (72.1%) were male. All patients had advanced inoperable tumors, in which 73 (34.4%) with stage IIIB, and 139 (65.6%) with stage IV disease. According to the cancer cell types, adenocarcinoma was the most common histology (n = 117, 55.2%), followed by 62 (29.2%) squamous cell carcinoma and 33 (15.6%) adenosquamocarcinoma. All patients had an ECOG performance status of 1 or 0. There were 120 (56.6%) smokers and 92 (43.4%) non-smokers. No patient has received definitive thoracic radiotherapy, whereas 38(17.9%) patients were radiated as palliative treatment to relieve symptoms caused by airway obstruction or pain by bone metastasis. The majority of the patients (n = 146, 68.9%) received 3 or more cycles of chemotherapy during the first-line treatment, while 66(31.1%) patients received 2 or less cycles of chemotherapy. Table 1 shows the clinical and pathologic characteristics of patients.

**Table 1:** Clinical characteristics of NSCLC patients.

Patient characteristics	Number (%)
Total no. patients = 212	
Median age 60 (33–77)	
Gender	
Male	153(72.1)
Female	59(27.9)
PS*	
0	34(16.0)
1	178(84.0)
Smoking status	
Non-smoker	92(43.4)
Smoker	120(56.6)
Stage	
IIIB	73(34.4)
IV	139(65.6)
Histological cell type	
Adenocarcinoma	117(55.2)
Squamous cell	62(29.2)
Adenosquamocarcinoma	33(15.6)
Radiation therapy	
Yes	38(17.9)
No	174(82.1)
Chemotherapy	
GP	133(62.7)
GC	17(8.1)
NP	26(12.3)
NC	16(7.5)
T/P+P	7(3.3)
T/P+C	13(6.1)
The cycles of regimens received	
1	3(1.4)
2	63(29.7)
3	28(13.2)
4	108(50.9)
5∼6	10(4.7)

GP = gemcitabine + cisplatin. GC = gemcitabine + carboplatin. NP = vinorelbine + cisplatin. NC = vinorelbine+ carboplatin. T/P+ P = docetaxel/paclitaxel+ cisplatin. T/P+ C = docetaxel/paclitaxel+ carboplatin.

* PS, Performance Status.

For the COX-2 A/G polymorphism at rs689465, 178(84.0%) patients were homozygous of the A/A genotype, whereas 31(14.6%) were heterozygous A/G and 3 (1.4%) were variant homozygotes G/G. For the COX-2 rs689466 polymorphism, 67 (31.6%) patients were homozygous of the A/A genotype, whereas 99(46.7%) were heterozygous A/G and 46 (21.7%) were variant homozygotes G/G. For the COX-2 rs3218625 polymorphism, 207 (97.6%) patients had the reference G/G genotype, whereas 5 (2.4%) were A/G. For the COX-2 rs20417 polymorphism, 195 (92.0%) patients were homozygous of the G/G genotype, whereas 16(7.5%) were heterozygous A/G and 1 (0.5%) were variant homozygotes C/C. All the genotype distributions were in Hardy-Weinberg equilibrium (P>0.05).

All patients received platinum-based chemotherapy. 150(70.8%) received gemcitabine plus cisplatin/carboplatin regimens (GP or GC), 42 (19.8%) received vinorelbine plus cisplatin/carboplatin (NP or NC) and 20(9.4%) were given docetaxel/taxel plus cisplatin/carboplatin (DP or DC; PP or PC). There was no significant association between the distribution of treatment regimens and polymorphism group. Table 2 shows the detailed distribution of treatment regimen by polymorphism group.

**Table 2:** Allele frequencies of the polymorphisms and the distribution of chemotherapy regimens by polymorphism group.

Genotype	Total (%)	GP(n)	GC	NP	NC	T/P+P	T/P+C
**rs689465**							
A/A	178(84.0)	115	12	22	13	6	10
A/G	31(14.6)	18	5	3	3	1	1
G/G	3(1.4)	0	0	1	0	0	2
**rs689466**							
A/A	67(31.6)	38	5	9	4	5	6
A/G	99(46.7)	64	8	12	8	2	5
G/G	46(21.7)	31	4	5	4	0	2
**rs20417**							
C/C	1(0.5)	1	0	0	0	0	0
C/G	16(7.5)	11	1	2	0	1	1
G/G	195(92.0)	121	16	24	16	6	12
**rs3218625**							
A/A	0(0)	0	0	0	0	0	0
A/G	5(2.4)	4	0	0	1	0	0
*G/G*	207(97.6)	133	17	26	16	7	13

All chemotherapy-related toxicities were recorded in each treatment cycle. Incidences of all grade 3 and 4 toxicities were shown in Table 3. Eighty-two patients (38.7%) suffered from grade 3 or 4 hematologic toxicity, of whom 58 (27.4%) had grade 3 or 4 leukopenia, 26 (12.3%) grade 3 or 4 thrombocytopenia, 8 (3.8%) grade 3 or 4 anemia. Twenty-eight (13.0%) patients experienced grade 3 or 4 gastrointestinal toxicity. Additionally, Twenty-three patients (10.8%) and ten patients (4.6%) suffered from alopecia (any grade) and 3 or 4 cardiac toxicity, respectively. There were no patients who experienced more than one category of the toxicities.

**Table 3:** Treated Patients with CTC Grade 3 or 4 Drug-Related Toxicities (worst grade)*.

Toxicity	N (%)
**Hematologic toxicity**	82(38.7)
Leukocytopenia	58(27.4)
Thrombocytopenia	26(12.3)
Anemia	8(3.8)
**Non-hematologic toxicity**	
Nausea/vomiting	28(13.0)
Alopecia, any grade	23(10.8)
Cardiac toxicity	10(4.6)

* Only toxicities reported in at least 3% of patients are listed.

### Association between COX-2 polymorphisms and grade 3 or 4 toxicity

Logistic regression was carried out to reveal the association between COX-2 polymorphisms and patient outcomes. The association between polymorphisms and toxicity was shown in Table 4.

**Table 4:** Association between COX-2 polymorphisms and 3 or 4 Drug-Related Toxicities (worst grade).

Genotype	Total	3 or 4 Hematologic toxicity	P	3 or 4 Leukopenia	P	3 or 4 Thrombocytopenia	P	3 or 4 Anemia	P	3 or 4 Nausea/vomiting	P	Alopecia	P	3 or 4 Cardiac toxicity	P
**rs689465**															
AA	178	70		53		19		6		22		19		8	
AG+GG	34	12	0.711	5	0.105	7	0.138	2	0.516	6	0.726	4	0.867	2	0.971
**rs689466**															
AA	67	17		10		8		1		9		6		3	
AG	99	43		33		11		5		15		11		4	
GG	46	22		15		7		2		4		6		3	
AG+GG	145	65	**0.008** *	48	**0.010** *	18	0.992	7	0.206	19	0.626	17	0.111	7	0.852
**rs20417**															
GG	195	76		54		24		7		24		21		9	
CG+CC	17	6	0.655	4	0.770	2	0.752	1	0.886	4	0.255	2	0.859	1	0.796
**rs3218625**															
GG	207	80		56		26		7		28		22		9	
AG+AA	5	2	0.811	2	0.524	0	1.000	1	**0.045^+^**	0	1.000	1	0.252	0	1.000

* Data were calculated by unconditional logistic regression. P value shown in the table was original P value.

* Bold, P-value was still significant after the Bonferroni correction, adjusted P value were 0.024 and 0.030, respectively. + Bold, no more significance after the Bonferroni correction, adjusted P value was 0.135.

The incidence of grade 3 or 4 hematologic toxicity (44.8%) was significantly higher in G allele carriers of the COX-2 rs689466 polymorphism (P = 0.008; OR = 2.47; 95%CI = 1.26–4.84; the significance remained after the Bonferroni correction (P = 0.024); Bold in Table 4.) compared to those with wild-type homozygotes AA (25.4%). When only severe leukopenia toxicity was considered, statistically significant difference was also found in rs689466 polymorphism carried with the frequency of different genotypes and occurrence of grade 3 or 4 leukopenia (P = 0.010; OR = 2.82;95%CI = 1.28–6.20;the significance remained after the Bonferroni correction (P = 0.030); Bold in Table 4). Analysis of grade 3 or 4 thrombocytopenia or anemia toxicity revealed no statistically significant association with rs689466 polymorphism. No other significant association between genotype and non-hematologic toxicity was observed in this polymorphism.

It should be noted that the variant AG and AA genotypes of rs3218625 polymorphism showed a tendency to be associated with high risk of anemia compared with the GG genotype (P = 0.045; OR = 16.55; 95%CI = 0.67–86.75), However, the significance lost after the Bonferroni correction (P = 0.135, Table 4). There was no other significant association between the risk of any grade 3 or 4 toxicity and rs689465 and rs20417 polymorphisms (Table 4).

### Haplotype analysis

Pairwise linkage disequilibriums for the four SNPs are presented respectively. rs689466, rs3218625 and rs20417 polymorphisms were in strong linkage disequilibrium with each other and therefore formed a haplotype block. The two most common haplotypes, AGG and GGG (in the order of rs689466, rs3218625 and rs20417), were found to account for 94.6% of the study population. Global score test showed statistically significant differences in haplotype frequency distribution and the occurrence of grade 3 or 4 hematologic and leukopenia toxicity (global X^2^ = 6.74, df = 1, P = 0.009, and Global X^2^ = 5.02, df = 1, p = 0.025, respectively, Bold in Table 5). The haplotype AGG was associated with a reduced risk of grade 3 or 4 hematologic and leukopenia toxicity (P = 0.009; OR = 0.59; 95%CI = 0.39–0.88 and P = 0.025; OR = 0.61; 95%CI = 0.39–0.94, respectively), while the haplotype GGG was associated with an increased risk of grade 3 or 4 hematologic and leukopenia toxicity (P = 0.009; OR = 1.71; 95%CI = 1.14–2.56 and P = 0.025; OR = 1.65; 95%CI = 1.06–2.57, respectively). No significant association between other haplotypes and 3 or 4 toxicities was observed (Table 5).

**Table 5:** COX-2 haplotypes and 3 or 4 Drug-Related Toxicities (worst grade).

Haplotypes*	A G C	p	A G G	p	G A G	p	G G C	p	G G G	p	Global score test
**Hematologic toxicity**											
3, 4 grade, n (%)	6(3.6)	NA	71(43.3)	**0.009**	2(1.2)	NA	0(0.0)	NA	85(51.8)	**0.009**	Global *X* ^2^ = 6.74, df = 1, **p = 0.009**
0, 1, 2 grade, n (%)	12(4.6)		144(55.4)		3(1.2)		0(0.0)		101(38.8)		
**Leukopenia**											
3, 4 grade	4(3.4)	NA	49(42.3)	**0.025**	2(1.7)	NA	0(0.0)	NA	61(52.6)	**0.025**	Global *X* ^2^ = 5.02, df = 1, **p = 0.025**
0, 1, 2 grade	14(4.5)		166(53.9)		3(1.0)		0(0.0)		125(40.6)		
**Thrombocytopenia**											
3, 4 grade	0(0.0)	NA	27(51.9)	0.981	0(0.0)	NA	2(3.8)	NA	23(44.3)	0.981	Global *X* ^2^ = 0.001, df = 1, p = 0.981
0, 1, 2 grade	16(4.3)		190(51.1)		5(1.3)		0(0.0)		161(43.3)		
**Anemia**											
3, 4 grade	1(6.2)	0.372	6(37.5)	0.281	1(6.2)	0.056	0(0.0)	NA	8(50.0)	0.614	Global *X* ^2^ = 4.50, df = 3, p = 0.212
0, 1, 2 grade	17(4.1)		209(51.2)		4(1.0)		0(0.0)		178(43.6)		
**Nausea/vomiting**											
3, 4 grade	4(7.1)	0.259	28(50.0)	0.834	0(0.0)	NA	0(0.0)	NA	24(42.8)	0.803	Global *X* ^2^ = 1.28, df = 2, p = 0.528
0, 1, 2 grade	14(3.8)		187(50.8)		5(1.4)		0(0.0)		162(44.0)		
**Alopecia**											
3, 4 grade	0(0.0)	NA	23(50.0)	0.925	1(2.2)	NA	2(4.3)	NA	20(43.5)	0.925	Global *X* ^2^ = 0.009, df = 1, p = 0.925
0, 1, 2 grade	16(4.2)		194(51.3)		4(1.1)		0(0.0)		164(43.4)		
**Cardiac toxicity**											
3, 4 grade	0.5(2.5)	NA	9.50(47.5)	0.736	0(0.0)	NA	0.5(2.5)	NA	9.50(47.5)	0.736	Global *X* ^2^ = 0.14, df = 2, p = 0.736
0, 1, 2 grade	17(4.2)		206(51.0)		5(1.2)		0(0.0)		176(43.5)		

Order of polymorphisms: rs689466, rs3218625, rs20417.

* Haplotypes were omitted if the estimated haplotype probability was less than 5%; Bold, P-value was significant. NA, not applicable.

## Discussion

In the present study, we investigated whether polymorphisms of COX-2 were associated with increased toxicity in advanced NSCLC patients treated with platinum-based chemotherapy. We found that patients carrying at least one variant COX-2 rs689466 G allele (AG or GG) were associated with a significantly increased risk of grade 3 or 4 hematologic and leukopenia toxicity.

COX-2 is an inducible form of the enzyme and expresses primarily in response to inflammatory stimuli like cytokines or growth factors and mediates the production of prostaglandins that support the inflammatory process [13]. In relation to radiation-induced oral mucositis, it has been demonstrated that COX-2 expression in hamsters increased their response to targeted radiation after radiation injury [29]. The expression of COX-2 is also associated with radiation-induced small bowel injury [30] and pulmonary inflammation [17]. In addition, selective COX-2 inhibitor may protect normal tissues by reducing acute inflammation and fibrosis when given with radiation [31], [32]. However, the association between the expression of COX-2 and chemotherapy-induced toxicity has not been reported yet.

Recent studies suggest that SNPs in COX-2 promoter may alter the enzyme function of COX-2 by differential regulation of COX-2 expression. The rs689466 polymorphism locates in the COX-2 promoter region, which contains several key cis-acting regulatory elements and has decisive roles in the regulation of COX-2 transcription [23], [33]. COX-2 rs689466 could affect gene transcription and mRNA stability, modulate inflammatory response and consequently contribute to individual variation in the susceptibility to cancers. Zhang et al. reported that rs689466 change created a transcriptional factor c-myeloblastosis (c-MYB) binding site and displayed a higher promoter activity. The rs689466 AA genotype significantly increased the expression of COX-2 mRNA levels compared with the GG genotype [14].

C-MYB is a transcriptional factor engaged in the hematopoietic system and plays an important regulatory role in cell growth, differentiation, malignant transformation and survival by targeting a variety of genes [34]. Studies show that the directive differentiation of erythroid,myeloid, megakaryocytic progenitors is related to the level of the expression of C-MYB [35]–[37].

Therefore, it is suggested that the sequence variation that creates the c-MYB binding site, such as COX-2 rs689466 polymorphism, may alter the level and specificity of gene transcription. We supposed that the association of COX-2 genotypes with increased risk of grade 3 or 4 hematologic and leukopenia toxicity might be attributed to gain of function of the gene resulting from the promoter SNPs. Another reasonable explanation is that rs689466 AA homozygous increases the expression of COX-2 mRNA and enhances the transcriptional activity of COX-2, thus causing reduction of apoptosis and further reduces the risk of severe toxicity, which has been confirmed by another study [6]. While the etiology and the mechanism of these outcomes are unknown, further experiments still need to reveal the detailed molecular mechanisms.

Previous clinical studies have suggested the expression of COX-2 as a predictive factor for survival in NSCLC patients [15], [38]. In the study by Edelman et al, patients with advanced NSCLC expressing moderate to high COX-2 protein levels had worse survival than those with lower expression levels [38]. Groen et al. reported that the high expression of COX-2 with better PS and adenocarcinoma was associated with better survival [39]. Few studies have been reported thus far regarding to the association between COX-2 rs689466 polymorphism and clinical outcomes in NSCLC patients. Nan Bi et al. examined five functional COX-2 polymorphisms and suggested that only COX-2 rs689466 polymorphism was a potential predictive marker for survival in locally advanced NSCLC patients treated with chemoradiotherapy or radiotherapy alone and the GA and GG genotypes were significantly correlated with better overall survival and with longer progress-free survival compared with the rs689466 AA genotype [20]. In addition, a recent study that included 231 patients with lung cancer also suggested that COX-2 rs689466 may be a risk factor for the development of lung cancer [40].

All the studies presented above, including ours, suggest that COX-2 rs689466 polymorphism plays an important role in cancer development and might be a useful molecular indicator of cancer risk, prognosis, response to treatment and toxicity.

However, our study has several limitations. First, treatment heterogeneity, i.e. different combination regimens and the duration of the therapy may influence the result. However, all the ORs and 95%CIs had been adjusted by types of treatment regimens, including cisplatin versus carboplatin, taxanes versus non-taxanes and the number of cycles of chemotherapy received during first-line treatment in logistic regression model, thus the potential confounding factors were minimized. Second, in our study, we found that COX-2 rs689466 variant A allele frequency was similar to those data that has been published in Chinese population [14], [20], but this SNP is ethnic specific compared with Caucasian [21], [40], thus our results should be validated among different ethnic populations. Finally, prior studies failed to show any significant association between COX-2 expression and toxicity. After reviewing these articles carefully, we consider that they mainly focused on the role of COX-2 in cancer development, epidemiology and predictive role for survival [12], [13], [15], [38], [39]. Instead, our study chose to only focus on drug-related toxicity, mainly hematologic toxicity, and the association between toxicity and COX-2 polymorphisms, not COX-2 expression. Further prospective validation studies should be carried out to replicate the findings.

Our study also has several strengths. We assess the grading of toxicities according to the National Cancer Institute Common Toxicity Criteria version 3.0 among clinicians comprehensively. A relatively large number of patients with advanced NSCLC receiving platinum-based chemotherapy were carried out independently without the knowledge of polymorphism status enrolled in this study. All patients were treated at the same hospital. It seems that our finding is not likely to have been obtained by chance.

To the best of our knowledge, this is the first study showing that the COX-2 polymorphism could predict toxicity outcomes in patients with advanced stage NSCLC treated with platinum-based chemotherapy. We found that the rs689466 polymorphism was associated with severe hematologic and leukopenia toxicity risk.

## Conclusion

This investigation for the first time suggested that polymorphism in COX-2 rs689466 may be a potent bio-marker in predicting severe hematologic toxicity in NSCLC patients after platinum-based chemotherapy.
